# Efficiency of Diagnostic Testing for *Helicobacter pylori* Infections—A Systematic Review

**DOI:** 10.3390/antibiotics10010055

**Published:** 2021-01-08

**Authors:** Paula Rojas García, Simon van der Pol, Antoinette D. I. van Asselt, Maarten Postma, Roberto Rodríguez-Ibeas, Carmelo A. Juárez-Castelló, Marino González, Fernando Antoñanzas

**Affiliations:** 1 Department of Economics and Business, University of La Rioja, 26004 Logroño, Spain; roberto.rodriguez@unirioja.es (R.R.-I.); carmelo.juarez@unirioja.es (C.A.J.-C.); marinojgonzalez@gmail.com (M.G.); fernando.antonanzas@unirioja.es (F.A.); 2 University Medical Center Groningen, Department of Health Sciences, University of Groningen, 9713 GZ Groningen, The Netherlands; s.van.der.pol@rug.nl (S.v.d.P.); a.d.i.van.asselt@umcg.nl (A.D.I.v.A.); m.j.postma@rug.nl (M.P.); 3 University Medical Center Groningen, Department of Epidemiology, University of Groningen, 9713 GZ Groningen, The Netherlands; 4 Department of Economics, Econometrics and Finance, University of Groningen, 9747 AE Groningen, The Netherlands

**Keywords:** *Helicobacter pylori*, diagnostic testing, antibiotics, systematic review, AMR

## Abstract

Background: The most recommended treatment for a *Helicobacter pylori* infection is high doses of combined antibiotics. The objective of this article is to perform a systematic review of the economic evaluation studies applied to assess the efficiency of diagnostic testing for *H. pylori* infections, so that their main characteristics can be identified and to learn from the literature how the antimicrobial resistance (AMR) issue is incorporated into these economic evaluations. Methods: We conducted a systematic review to compare the costs and clinical effectiveness of diagnostic strategies for *H. pylori* infections. We followed the Preferred Reporting Items for Systematic Reviews and Meta-Analyses (PRISMA) guidelines and extracted the items from the Consolidated Health Economic Evaluation Reporting Standards (CHEERS) checklist. Results: We found thirteen articles that were of good quality according to CHEERS: six studies focused on diagnostics of *Helicobacter pylori* infections associated with dyspepsia and four on duodenal ulcers. Testing was found to be the most cost-effective strategy in eight articles. Four studies considered AMR. Conclusions: Testing was more cost-effective than empirical treatment, except in cases of high prevalence (as with developing countries) or when patients could be stratified according to their comorbidities. The introduction of AMR into the model may change the efficiency of the testing strategy.

## 1. Introduction

*Helicobacter pylori* (hence forth referred to as *H. pylori)* infection affects over half the world’s population [1]. As described by Warren and Marshall in 1983 [2], this infection has been associated with disorders such as peptic ulcers, chronic gastritis, dyspepsia, lymphomas of lymphoid tissue of the gastric mucosa and gastric cancer [3,4,5]. *H. pylori* has been reported to cause 90% of duodenal ulcers and 80% of gastric ulcers [6].

The frequency of *H. pylori* infection and its consequences has influenced the definition of treatment standards. The V Maastricht Consensus for the Treatment of *H. pylori* Infections (2015) [7] recognizes the implications that antimicrobial resistance has had on the effectiveness of treatments. The Consensus notes the increasing rates of resistance in high and middle-income countries. Levels of resistance to clarithromycin reach 30% in Italy and Japan, 40% in Turkey and 50% in China, among others [8,9,10,11,12,13]. Therefore, the Consensus recommends that standard triple therapy (the combination of PPI (proton pump inhibitor)-clarithromycin and amoxicillin or metronidazole) without prior susceptibility testing should not be used when resistance to clarithromycin exceeds 15%. Furthermore, another cause of reduction in the eradication rate is the presence of biofilms on the surface of gastric mucosa, which may cause antibiotic treatment to fail. As noted in the literature, *H. pylori* biofilm formation increases the threat of antimicrobial resistance (AMR) development [14].

At present, the adequate treatment of *H. pylori* infections requires progress in two areas: improving the quality of existing or new diagnostic tests so that infections are identified more quickly and accurately [15,16,17] and widening the diagnostic options to detect better AMR before treatment is prescribed.

Non-invasive and invasive methods are currently available for diagnosing *H. pylori* [1,18]. Most frequently included among the former are the urea breath test (UBT) and the stool antigen test. The invasive diagnostic option is the upper endoscopy, including histological testing, polymerase chain reaction (PCR), culture and rapid urease testing (RUT). PCR tests have been proposed as one of the diagnostic alternatives to avoid endoscopies and to evaluate bacterial resistance. It has been reported that the Amplidiag *H. pylory*+ClariR Mobidiag essay has a high sensitivity and specificity for the detection of both *H. pylori* and CLA resistance [19].

Evidence of the role of antimicrobial resistance in reducing the rate of eradication influences the use of other therapeutic options, such as bismuth quadruple therapy, quadruple sequential therapy, quadruple concomitant therapy (QCT) and hybrid therapy [20]. It has been reported that QCT may overcome the declining *H. pylori* eradication rate [20]. Although quadruple-regimen therapy (bismuth or non-bismuth) has been reported to be useful when resistance to clarithromycin or metronidazole is present, it also increases resistance if treatment is prolonged with multiple antibiotics [21].

The worrying evolution of the increase in AMR, including primary resistance, has generated a growing international consensus on the importance of tailored therapy through analysis of susceptibility prior to the initiation of treatment for *H. pylori* infection [6,21,22]. However, susceptibility testing is not commonly performed [22]. The high frequency of this infection results in the use of primary care services, causing indications of antibiotics and increasing the chances of antimicrobial resistance. That is why it is particularly important to analyze the economic evaluation of diagnostic alternatives in these diseases that will facilitate the adoption of evidence-based decision strategies regarding antibiotic treatments and, consequently, the potential reduction of AMR. We are particularly interested in the studies that examine the existence of AMR and its effects on the efficiency of antibiotic treatment.

The objective of this article is twofold. First, we perform a systematic review of the economic evaluation studies applied to assess the efficiency of diagnostic testing for the *H. pylori* infection. We intend to summarize the methods applied to these economic evaluations and to highlight the main characteristics of these studies. The second objective is to learn from the literature review how the AMR issue is incorporated in economic evaluation of diagnostic testing.

## 2. Materials and Methods

### 2.1. Types of Studies

The Preferred Reporting Items for Systematic Reviews and Meta-Analyses (PRISMA) guidelines were followed in this study [23]. Articles included in this review compare both the costs and the clinical effectiveness outcomes of at least two different diagnostic strategies for *H. pylori* infection. We assess the efficiency a strategy that reduces the uncertainty of the physician when a patient with symptoms common to several diseases must be diagnosed in clinical practice. Accordingly, screening and genotype studies were excluded. The main difference between diagnostics and screening is the population: in the former it concerns patients presenting with symptoms and in the latter it concerns the general population which is to be healthy [24,25]. Protocols and review articles were also excluded.

### 2.2. Search Strategy and Selection Criteria

The syntax used in the search was created to retrieve economic evaluations of diagnostic strategies for the management of *H. pylori* infection (Appendix A). Articles included in this systematic review were obtained from three databases of peer-reviewed literature: Scopus, PubMed and Web of Science. Geographical limitations were not established but in order to provide updates on clinical practice, only articles published between January 2000 and October 2020 were included. The first round consisted of title and abstract screening performed by P.R.G., M.G., R.R.I., C.A.J.C. and S.v.d.P. Duplicates were removed and articles were selected according to the aforementioned inclusion criteria. In the second step, full-text reports were evaluated for eligibility. In case of any discrepancy among the reviewers, another reviewer was asked (A.D.I.v.A.).

### 2.3. Data Extraction and Analysis

In order to obtain the data from the included articles, authors followed the Consolidated Health Economic Evaluation Reporting Standards (CHEERS) checklist, performing consistency checks as recommended [26]. Furthermore, new items not considered in the CHEERS checklist were added: AMR included in the model, specific limitation found relating to the diagnostic strategy and the pros and cons of the modelling technique identified by the authors. Microsoft Excel was used to manage data extraction and categorize articles by the management of infection. This software was also used to transform data and create tables. The references manager Zotero was used to store the bibliography.

## 3. Results

**Figure 1:**
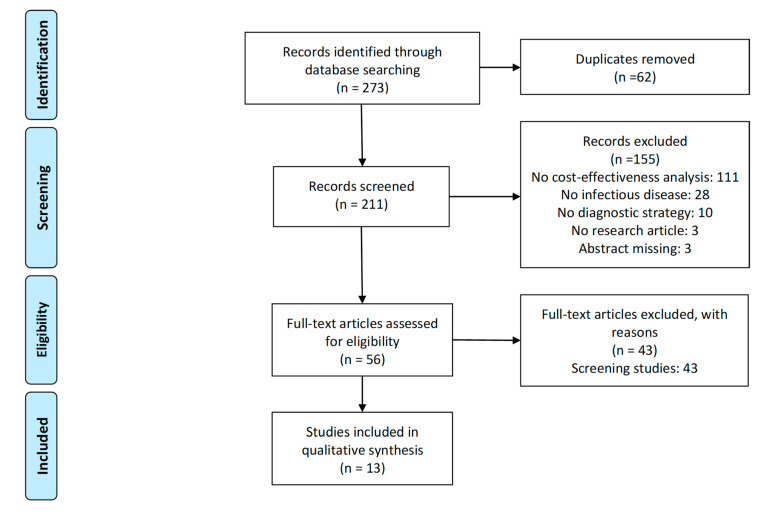
PRISMA flow diagram.

A total of thirteen articles were retrieved through the systematic review. Figure 1 shows a PRISMA flow diagram of inclusion and exclusion number of articles. According to the abstract of these articles, we have classified the studies into three groups: diagnostics of *H. pylori* infection associated with dyspepsia, diagnostics associated with duodenal ulcers and diagnostics associated with other symptoms.

Table 1 shows the quality of the articles, in terms of the items reported and recommended in the CHEERS checklist. Most items were found in the articles. Most of the articles had a time horizon of less than one year, so it was not necessary to report discount rates.

### 3.1. Diagnostics of H. pylori Infection Associated with Dyspepsia

Six articles [27,28,29,30,31,32] examined the cost-effectiveness of a range of test and treat strategies to manage patients attending primary care with dyspepsia as the predominant symptom. Table 2 shows the models’ main characteristics. Two models [30,32] introduced AMR into the analysis: reducing the eradication rate for triple therapy (ranitidine, metronidazole and tetracycline) from 80–100% to 50–100%, arguing that as in China over-the-counter antibiotics are occasionally available, AMR may cause a higher failure rate [30] and reducing the eradication rate, as the prevalence of clarithromycin resistance increases [32]. All articles assess the use of a *H. pylori* test and in four of them this was found to be the most cost-effective strategy. In one of the other two cases, the most cost-effective strategy was to stratify patients using a score system (using a previously validated predictive model) then referring those at higher risk of organic dyspepsia to endoscopy [29]. In the other one, treating them with empiric PPI even when the prevalence of *H. pylori* infection varied from 5% to 40% [31]. This last result was reached after authors modelled how the test is actually used in U.S. practice, assuming that clinicians would perform a biopsy in the case of a lack of symptomatic relief, thus reducing the benefits of testing.

### 3.2. Diagnostics of H. pylori Infection Associated with Duodenal Ulcers

Four articles [33,34,35,36] studied the cost-effectiveness of alternative strategies of diagnosing *H. pylori* infection in patients with duodenal ulcers. Table 3 shows the main characteristics of the models. In two articles [34,35] empirical triple therapy was the most cost-effective approach, considering that the analysis was performed in a country with high prevalence of the infection and first-line therapy was more cost-effective than treatment for recurrent ulcers or long-term maintenance treatment. One model [36] introduced AMR into the analysis, taking into consideration that diagnostic testing can provide rapid and reliable results regarding the presence of clarithromycin resistance. The dual priming oligonucleotide (DPO) PCR test, which gives information regarding clarithromycin resistance, reduced secondary prescriptions, thus making this strategy more cost-effective than other diagnostic approaches, such as rapid urease tests.

### 3.3. Diagnostics of. H. pylori Infection

Three articles [37,38,39] studied the cost-effectiveness of alternative initial strategies of diagnosing *H. pylori* infection in patients attending primary care with any predominant symptom. Table 4 shows the models’ main characteristics. Two studies [37,39] found that the initial test for *H. pylori* was the most cost-effective strategy, although this result depended on the prevalence of the *H. pylori* infection. The other article [38] introduced AMR into its analysis, considering that, if the first antibiotic treatment failed due to clarithromycin-resistance, the patient was treated with metronidazole. In this case, testing for *H. pylori* was not cost effective in the given modest prevalence of clarithromycin resistance. When the model considered a high prevalence of clarithromycin resistance (>45%), testing was the most cost-effective alternative.

## 4. Discussion

In the present literature review, thirteen articles related to the efficiency of diagnostic testing for *H. pylori* infection were retrieved following the PRISMA guidelines. All of them are of a good quality in terms of the items reported and recommended in the CHEERS checklist. A preferable strategy should reflect a long-term time horizon (as *H. pylori* infections can lead to other health conditions), include AMR in the analysis (which could be as simple as reducing the disease eradication rate based on the prevalence of resistant infections in the population) and report time or costs until correct diagnosis or appropriate treatment prescribed (due to the high antibiotic doses that the treatment of *H. pylori* demands). As new and faster diagnostic tests become available, economic evaluations should be used to assess their cost-effectiveness.

This review represents a small number of studies. In the 1990s, the diagnosis of *H. pylori* infection was based on invasive approaches such as endoscopy [40]. Eight articles were published between 2000 and 2010, highlighting the rise of the breath test, which led to a proliferation of these studies. The current literature focuses on the increasing demand for rapid non-invasive tests that can inform prescriptions. The standard treatment for *H. pylori* infection is based on high doses of combined antibiotics and second-line antibiotics with more risk of AMR [15]. The most recently published articles consider AMR in their analysis, decreasing the eradication figures [30,32,38]. In these articles, the study is performed in a country where antibiotics can be occasionally obtained over the counter (year 2006, China) or in high-resistance areas (Greece and Japan), leading to AMR causing a higher failure rate. Even in other countries where antibiotics are only given with a prescription, the rate of patients that present resistance to antibiotics is increasing, worsening this public health threat [41]. The inclusion of AMR into the analysis has been done by reducing the rate of antibiotic efficacy but other alternatives such as increasing treatment costs have not been found in any article. Interestingly, AMR can change the results of the most cost-effective strategy to diagnose *H. pylori* infections [38]. Furthermore, the CHEERS checklist focused on methodological issues, thus the inclusion of AMR is not considered as an important item to be reported.

The lack, or even delay, of diagnostic tests for the *H. pylori* infection increases the risk of developing not only AMR but also significant complications [17,42]. In this review, nine articles reported a time horizon of one year or shorter. Only three articles used a longer time horizon in order to capture the medical consequences of developing gastric cancer [31], acute treatment failure [43] or childhood acute lymphoblastic leukemia [44]. The reviewed articles did not consider either the relapses or biofilm formation, perhaps due to the short time horizon reported in the majority of the studies. Future studies could be extended using other forms of modeling. In this review, most of the articles (78%) used a decision tree to determine the cost-effectiveness of different diagnostic techniques. Only three articles performed a Markov model and two of them [31,44] considered an extended time horizon. The most frequent clinical outcome reported was cost or length of time until correct diagnosis [32,33,37,38,39,45], cost per appropriate treatment prescribed [27,30,34,45] and second-line antibiotics treatment safely avoided [36], which highlights the importance of a correct diagnosis to reduce unnecessary antibiotic treatment. Also, quality adjusted life year (QALYs) [35,44] and days free from disease [28,29,31] were used.

As a limitation, this review specifically excludes screening studies, which are carried out in some regions due to the higher prevalence of *H. pylori* infections in older patients, as an effect of a generation exposed to poor sanitation [46]; the number of articles finally selected was therefore substantially reduced. However, we wanted to assess the cost-effectiveness of a testing strategy when the patient has at least one related symptom of infection. Screening studies do not take any previous symptoms into consideration. Setting was not reported particularly in articles of *H. pylori* infection associated with dyspepsia or duodenal ulcers, discerning between screening and diagnostics strategies was not straightforward. In all cases, the reviewers agreed on inclusion, if the article assessed a diagnostic test or exclusion, if the article considered a screening strategy. The number of studies found was geographically limited and, apart from Spain and Greece, there were no studies from other European countries. It would be interesting to know the efficiency of this diagnostic approach in regions other than the USA and Asia, in order to select the most appropriate for *H. pylori* infection. Finally, although we limited the published year to include last two decades studies, we believe that this period is wide enough to capture the time when the economic evaluation of this issue has been performed.

At this point, it is interesting to know how economic evaluation has been applied to the *H. pylori* infection so that the results on the efficiency of the different options can guide the adoption of decisions related to testing strategy. As with any other diagnostic technology, its efficiency is subject to the assumptions made on subsequent treatments; furthermore, given that these treatments are based on antibiotics, the considerations about the potential generation of resistances and its costs may drastically change the indication of the technology’s ultimate efficiency. How all these aspects can be accounted for in different studies is an interesting issue to be analyzed and included in future research.

## Figures and Tables

**Table 1:** CHEERS checklist results (percentage of articles that included the item).

CHEERS Items	*H. pylori* Infection Associated with Dyspepsia (n = 6)	*H. pylori* Infection Associated with Duodenal Ulcer (n = 4)	*H. pylori* Infection Alone (n = 3)
Title	100%	100%	100%
Abstract	100%	100%	100%
Background and objective	100%	100%	100%
Target population	100%	100%	100%
Setting	16%	25%	100%
Study perspective	100%	100%	100%
Interventions compared	100%	100%	100%
Treatment	100%	100%	33%
Time horizon	16%	100%	33%
Discount rate for health outcomes	0%	0%	0%
Discount rate for economic outcomes	0%	25%	0%
Reported clinical outcomes	100%	100%	100%
Measurement of effectiveness	100%	100%	100%
Resource and cost estimations	100%	100%	100%
Currency year used	50%	25%	33%
Type of model	100%	100%	100%
Assumptions	100%	100%	100%
Analytical methods	100%	100%	100%
Study parameters	100%	100%	100%
Characterizing uncertainty	100%	100%	100%
Study findings, limitations, generalizability and current knowledge	83%	100%	100%
Source of funding	83%	0%	33%
Conflicts of interest	50%	0%	33%

**Table 2:** Articles related to diagnosing *H. pylori* infection associated with dyspepsia.

First Author (year)	Country	Setting	Perspective and Time Horizon	Type of Model	Strategies Compared ^1^	Treatment	AMR Included	Uncertainty Reported
Chey (2001) [27]	USA	PC	Healthcare center’s— NA	Decision tree	(1) Antibody test, if positive treat; (2) **Active *H. pylori* infection test, if positive treat**	Lansoprazole, clarithromycin and amoxicillin	No	SAG
Makris (2003) [28]	Canada	PC	Healthcare payer’s— 1 year	Decision tree	(1) Empirical eradication therapy; (2) Endoscopy; (3) Barium examination; (4) Eradication therapy; (5) Antisecretory regimen; (6) UBT; (7) Laboratory testing, if positive therapy; (8) ***H. pylori* test and urea breath test**	Eradication therapy	No	DSA, tornado diagram, two-way SAG
García- Altés (2005)[29]	Spain	PC	Healthcare payer’s— 1 year	Decision tree	(1) Endoscopy; (2) **Score and scope**; (3) Test and scope; (4) Test and treat; (5) Empirical antisecretory treatment	Clarithromycin, amoxicillin and omeprazole	No	DSA, two-way SAG
You (2006) [30]	China	PC	Healthcare center’s— 1 year	Markov model	(1) Treat none; (2) Empirical PPI therapy; (3) **Test and treat**; (4) Endoscopy	Eradication therapy or PPI	Yes	DSA
Holmes (2010) [31]	USA	PC	Societal-lifetime	Markov model	(1) *H. pylori* tests; (2) *H. pylori* IgG test; (3) Stool antigen test; (4) IgG test; (5) UBT; (6) **PPI trial**	Eradication therapy or PPI	No	PSA
Papaefthymiou (2020) [32]	Greece	Hospital	Healthcare payer’s— 1 year	Decision tree	(1) Esophagogastroduodenoscopy; (2) **Specific UBT test for *H. pylori***; (3) Giemsa stain	Non-bismuth quadruple eradication	Yes	DSA

^1^ the most cost-effective strategy is in bold; PC, primary care; NA, not reported; PPI, proton pump inhibitor; DSA, deterministic sensitivity analysis; PSA, probabilistic sensitivity analysis; AMR, antimicrobial resistance; UBT, urea breath test; SAG, sensitivity analysis graph.

**Table 3:** Articles related to diagnosing *H. pylori* infection associated with duodenal ulcers.

First Author (year)	Country	Setting	Perspective and Horizon	Type of Model	Strategies Compared ^1^	Treatment	AMR Included	Uncertainty Reported
Rich (2000) [33]	USA	NA	Healthcare payer’s—1 year	Decision tree	(1) **Test and treat**; (2) Upper gastrointestinal radiography	Antibiotics and antisecretory agents	No	SAG
Ghoshal (2002) [34]	India	PC	Healthcare payer’s—1 year	Decision tree	(1) Anti-secretory therapy; (2) RUT and histological examination for ***H. pylori***; (3) **Empirical triple therapy**	Antisecretory, amoxycillin and tinidazole or PPI	No	Two-way SAG
Ghoshal (2003) [35]	India	Hospital	Healthcare payer’s—2 years	Decision tree	(1) Anti-secretory therapy; (2) RUT and histological examination for ***H. pylori***; (3) **Empirical triple therapy**	Antisecretory, amoxycillin and tinidazole or PPI	No	DSA, two-way SAG
Cho (2019) [36]	Korea	Hospital	Healthcare payer’s—1 year	Decision tree	(1) RUT; (2) **DPO-PCR**	Triple regimen or quadruple regimen	Yes	SAG, CE acceptability curve

^1^ the most cost-effective strategy is in bold; PC, primary care; NA, not reported; PPI, proton pump inhibitor; DSA, deterministic sensitivity analysis; AMR, antimicrobial resistance; RUT, rapid urease test; SAG, sensitivity analysis graph; DPO-PCR, dual priming oligonucleotide-based multiplex polymerase chain reaction.

**Table 4:** Articles related to diagnosing *H. pylori* infection with other symptoms.

First Author (year)	Country	Setting	Perspective and Horizon	Type of Model	Strategies Compared ^1^	Treatment	AMR Included	Uncertainty Reported
Vakil (2000) [37]	USA	PC	Healthcare payer’s—NA	Decision tree	Thirty-six testing strategies, included sequences of: **test for *H. pylori***, serology ELISA, UBT, fingerstick blood test, stool antigen test, RUT and histology	NA	No	SAG
Omata (2017) [38]	Japan	PC	Societal—1 year	Decision tree	(1) **RUT**; (2) Histology; (3) **Bacterial culture**; (4) Serum *H. pylori* IgG antibody (SHPAb); (5) UBT; (6) SHPAg; (7) UHPAb	Lansoprazole, amoxicillin and clarithromycin	Yes	SAG, CE acceptability curve
Beresniak (2020) [39]	Spain	PC	Healthcare system’s—1 year	Decision tree	(1) **Test and treat for *H. pylori***; (2) UBT; (3) Endoscopy; (4) Symptomatic treatment	Antibiotics (1st and 2nd line)	No	PSA

^1^ the most cost-effective strategy is in bold; PC, primary care; NA, not reported; DSA, deterministic sensitivity analysis; PSA, probabilistic sensitivity analysis; AMR, antimicrobial resistance; UBT, urea breath test; SAG, sensitivity analysis graph; ELISA, enzyme-linked immunosorbent assay; RUT, rapid urease test; SHPAb, serum *H. pylori* IgG antibody; UHPAb, urine *H. pylori* IgG antibody; CE, cost-effectiveness.

## Data Availability

Data is contained within the article.

## Appendix A

Syntax used in the search to retrieve economic evaluation of diagnostic strategies for the management of *H. pylori* infections.

**SCOPUS**
(TITLE-ABS-KEY(pharmacoeconomic *)
OR TITLE-ABS-KEY(cost-effectiveness)
OR TITLE-ABS-KEY(“economic evaluation”)
OR TITLE-ABS-KEY(“health technology assessment”))
AND (TITLE-ABS-KEY(antibiotic*)
OR TITLE-ABS-KEY(infectious)
OR TITLE-ABS-KEY(“bacterial infection”)
OR TITLE-ABS-KEY(“viral infection”))
AND (TITLE-ABS-KEY(“diagnostic”)
OR TITLE-ABS-KEY(“diagnostics”)
OR TITLE-ABS-KEY(“test”)
OR TITLE-ABS-KEY(“tests”)
OR TITLE-ABS-KEY(“testing”))
AND (TITLE-ABS-KEY(“pylori”))
AND PUBYEAR > 1999
AND PUBYEAR < 2020
**PUBMED**
(infectious
OR “bacterial infection”
OR “viral infection”
OR antibiotic *
OR antimicrobial)
AND (“diagnostic”
OR “diagnostics”
OR “test”
OR “tests”
OR “testing”)
AND (“1 Januray 2000”[Date—Publication]: “31 December 2020”[Date—Publication])
AND (pharmacoeconomic *
OR “cost-effectiveness”
OR “economic evaluation”
OR “health technology assessment”)
AND (“pylori”)
**WEB OF SCIENCE**
TS = (((“bacterial infection”
OR “viral infection”
OR antibiotic *
OR antimicrobial
OR infectious)
AND (“diagnostics”
OR “diagnostic”
OR “test”
OR “tests”
OR “testing”)
AND
(pharmacoeconomic*
OR cost-effectiveness
OR “economic evaluation”
OR “health technology assessment”)
AND (“pylori”)))
Period of time: 2000–2020
